# Risk factors for perioperative acute heart failure in older hip fracture patients and establishment of a nomogram predictive model

**DOI:** 10.1186/s13018-023-03825-2

**Published:** 2023-05-10

**Authors:** Miao Tian, Wenjing Li, Yan Wang, Yunxu Tian, Kexin Zhang, Xiuting Li, Yanbin Zhu

**Affiliations:** 1 Department of Orthopedic Surgery, The 3rd Hospital of Hebei Medical University, Shijiazhuang, 050051 Hebei People’s Republic of China; 2 Hebei Orthopedic Research Institute, Key Laboratory of Biomechanics of Hebei Province, Shijiazhuang, 050051 Hebei People’s Republic of China

**Keywords:** Heart failure, Hip fracture, Older people, Prediction model, Nomogram

## Abstract

**Aim:**

This study aims to explore the risk factors for perioperative acute heart failure in older patients with hip fracture and establish a nomogram prediction model.

**Methods:**

The present study was a retrospective study. From January 2020 to December 2021, patients who underwent surgical treatment for hip fracture at the Third Hospital of Hebei Medical University were included. Heart failure was confirmed by discharge diagnosis or medical records. The samples were randomly divided into modeling and validation cohorts in a ratio of 7:3. Relevant demographic and clinic data of patients were collected. IBM SPSS Statistics 26.0 performed univariate and multivariate logistic regression analysis, to obtain the risk factors of acute heart failure. The R software was used to construct the nomogram prediction model.

**Results:**

A total of 751 older patients with hip fracture were enrolled in this study, of which 138 patients (18.37%, 138/751) developed acute heart failure. Heart failure was confirmed by discharge diagnosis or medical records. Respiratory disease (odd ratio 7.68; 95% confidence interval 3.82–15.43; value of *P* 0.001), history of heart disease (chronic heart failure excluded) (odd ratio 2.21, 95% confidence interval 1.18–4.12; value of *P* 0.010), ASA ≥ 3 (odd ratio 14.46, 95% confidence interval 7.78–26.87; value of *P* 0.001), and preoperative waiting time ≤ 2 days (odd ratio 3.32, 95% confidence interval 1.33–8.30; value of *P* 0.010) were independent risk factors of perioperative acute heart failure in older patients with hip fracture. The area under the curve (AUC) of the prediction model based on these factors was calculated to be 0.877 (95% confidence interval 0.836–0.918). The sensitivity and specificity were 82.8% and 80.9%, respectively, and the fitting degree of the model was good. In the internal validation group, the AUC was 0.910, and the 95% confidence interval was 0.869–0.950.

**Conclusions:**

Several risk factors are identified for acute heart failure in older patients, based on which pragmatic nomogram prediction model is developed, facilitating detection of patients at risk early.

## Introduction

Heart failure (HF) is a common perioperative complication in older people with hip fracture, and it is also the second leading cause of in-hospital death, with an incidence ranging from 5.5 to 21.3% [1, 2]. The number of operations for hip fracture patients has increased consistently worldwide during the past decades [3–6]. The harmful effects of perioperative heart failure make it an important problem in the healthcare system. Previous studies showed that perioperative acute heart failure (AHF) substantially increased mortality within 30 days after surgery to 65%, prolonged the average length of stay of patients by 4 days, and increased the average hospitalization cost by about 5500 euros [7–10].

At present, many scholars pay more attention to AHF, which has lead to a significantly improved understanding of its etiology. Cardiac history, age, anemia, and ASA score have been shown to be risk factors for AHF [11–15]. However, these studies only obtained single and scattered risk factors for HF, which were unable to be translated into risk scales or prediction models. The New York Heart Association cardiac function rating scale and Goldman’s cardiac risk index (GCRI) were commonly used cardiac function assessment scales, but were not perfect. The first is a simple way to give scores according to patient complaints, which thus is easily affected by patients' subjective feelings and clinicians' subjective judgment, so it is somewhat biased when grading patients with mild heart failure [16, 17]. GCRI can evaluate the risk of perioperative cardiac complications, but it lacks model validation [18, 19]. The prediction model can integrate and quantify various risk factors, which can help medical personnel to individualize stratification and risk. Furthermore, although these two models have some practicability, none of them was specifically designed specifically for the assessment of older patients with hip fracture.

Given the high incidence of AHF in older patients with hip fractures, it has become increasingly necessary to establish a prediction model for AHF. Therefore, we designed this study to explore the risk factors for perioperative AHF in old patients with hip fracture and to build a nomogram model.

## Methods

### Study design and study population

In this study, data of older patients who underwent hip fracture surgery at our hospital from January 2020 to December 2021 were retrospectively collected. All included patients were 60 years or older and received surgical treatment for hip fractures. Exclusion criteria were: (1) patients with chronic heart failure; (2) missing data; (3) old fracture (21 days after injury); (4) multiple fractures; (5) pathological fractures. This study followed the Declaration of Helsinki and the protocol was approved by the Ethics Committee of the Third Hospital of Hebei Medical University with approval number 2022-014-1. This study is a retrospective study, so informed consent of patients was obtained by phone. Orthopedics, internal medicine physicians, and geriatric specialists jointly treat patients. By combining the patient's condition and examination results, the group discussed and decided on the infusion plan and treatment plan during the perioperative period.

### Definition of heart failure

In this retrospective study, the investigators reviewed the medical records to determine whether patients had perioperative AHF. The diagnostic criteria for AHF refer to the 2021 European Society of Cardiology guidelines for the diagnosis and treatment of acute and chronic heart failure [20]. The diagnosis of AHF was based on clinical symptoms (dyspnea, lung rales, lower limb edema, and rapid heart rate), laboratory examinations, and imaging examinations. At the same time, B-type natriuretic peptide (BNP) and cardiac troponin I (cTn I) should be considered together.

### Research methods

Two researchers collected 34 variables from demographic variables, operation-related variables, and laboratory parameters. Demographic variables included the gender of the patients, age, body mass index (BMI), fracture site (intertrochanteric fracture or femoral neck fracture), injury mechanism (low-energy or high-energy), the time from injury to admission, number of complications, comorbidities (anemia, hypertension, diabetes, cerebrovascular disease, etc.). Operation-related variables included the American Society of Anesthesiologists (ASA) classification, anaesthesia type, operation time, etc. Laboratory parameters included hemoglobin (HB), serum potassium concentrations, BNP, cTn I, etc. BMI was calculated by dividing the weight by height squared. According to the patient’s physical condition and the risk of surgery, the ASA classification divides patients into grades 1–5 (grade I: patients could tolerate the procedure well; grade II: patients had the mild systemic disease but no dysfunction; grade III: The patient had the severe systemic disease and certain dysfunction; grade IV: patients had the severe systemic disease and high anesthesia risk; grade V: dying patients).

To guarantee the homogeneity of the research objects, the researchers strictly implemented the inclusion and exclusion criteria. After two researchers inputted data, all data were cross-checked by a consultant researcher, also a researcher of this study. For suspicious or inconsistent data, the researchers corrected it by again referring to the medical records.

### Statistical analysis

In this study, IBM SPSS statistics 26.0 software was used for statistical analysis. Continuous variables were described as means ± standard deviation ($\overline{X}$ ± SD), and categorical variables were displayed as rates or percentages. Two independent sample *t*-tests or the Mann–Whitney *U* test was used to compare differences between groups for continuous variables, while the Chi-square test or Fisher exact test was performed for categorical variables.

The significance threshold was established at *P* < 0.05. Variables with a value of *P* < 0.05 in the univariate analysis were candidate variables in the multivariate models. Univariate and multivariate analyzes were conducted to determine the independent risk factors. The prediction efficiency of the model was analyzed using the receiver operating characteristic (ROC) curve. The Hosmer–Lemeshow test was used to evaluate the goodness-of-fit of the prediction model, and *P* > 0.05 indicated an accepted fitness. We validated the model using an internal data set. The predictors that were significantly associated with AHF were entered into the R software for the construction of the nomogram.

## Result

### Baseline characteristics

**Fig. 1:**
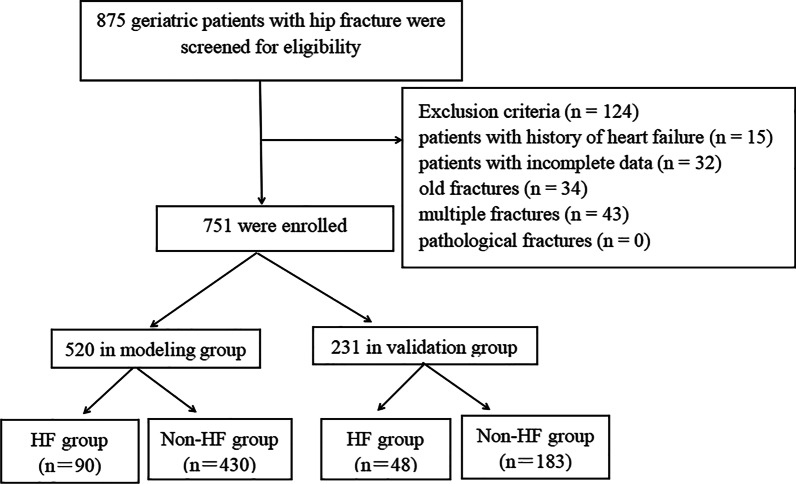
The flowchart showing the selection of research participants

As shown in Fig. 1, a total of 751 patients were included in this study, of which 520 were included in the modeling group using a simple random sampling method in a proportion of 7:3. Among the modeling group, 90 were patients with AHF and 430 were non-AHF patients. There were 138 patients (18.37%, 138/751) who developed AHF, with 3.86% for preoperative AHF and 14.51% for postoperative AHF. As shown in Tables 1 and 2, we can see that age, age-adjusted Charlson comorbidity index (ACCI), fracture type, preoperative waiting time, respiratory disease, acute kidney injury, history of heart disease (chronic heart failure excluded), anemia at admission, ASA ≥ 3, left ventricular ejection fraction (LVEF), BNP value at admission, Hb value at admission, blood transfusion before the operation, and serum potassium value at admission were statistically significant factors (*P* < 0.05). Given that intraoperative variables were not statistically significant in the univariate analysis (*P* > 0.05), their impact on the logistic multivariate regression analysis and the construction of the prediction model can be overlooked. Therefore, we did not further stratify our analysis by the occurrence time of heart failure and classified perioperative AHF into preoperative AHF and postoperative AHF. The result of multivariate analysis showed respiratory diseases (OR 7.68, *P* = 0.001), history of heart disease (chronic heart failure excluded) (OR 2.21, *P* = 0.010), preoperative waiting time ≤ 2 days (OR 3.32, *P* = 0.010), and ASA class ≥ 3 (OR 14.46, *P* = 0.001) were independent risk factors (Table 3). The ROC analysis showed that the AUC of age, Hb value at admission, BNP value at admission, LVEF, and ACCI were 0.595, 0.420, 0.621, 0.519, and 0.619. respectively. The cut-off values were 76.0 years old, 75.25 g/L, 32.50 pg/NL, 73.50%, and 4.50, respectively (Table 4).

**Table 1:** The characteristics of modeling group at admission

Variables	Heart failure group	Non­heart failure group	*P*
Age (years)	80.9 ± 6.9	78.5 ± 7.6	0.005
BMI (kg/m^2^)	23.3 ± 4.1	23.9 ± 3.9	0.174
ACCI	5.1 ± 1.2	4.5 ± 1.3	0.001
*Gender, n (%)*			0.887
Male	23 (25.6)	113 (26.3)	
Female	67 (74.4)	317 (73.7)	
*Fracture site, n (%)*			0.031
Intertrochanteric fracture	55 (61.1)	209 (48.6)	
Femoral neck fracture	35 (38.9)	221 (51.4)	
*Injury mechanism, n (%)*			
Low energy damage	86 (95.6)	415 (96.5)	0.896
High energy damage	4 (4.4)	15 (3.5)	
*The time from injury to admission, n (%)*			0.796
≤ 1 day	65 (72.2)	298 (73.3)	
1 day–7 days	20 (22.2)	88 (19.8)	
> 7 days	5 (5.6)	37 (7.0)	
Number of complications ≥ 3, *n* (%)	46 (51.1)	172 (40.0)	0.052
Anemia, *n* (%)	45 (50.0)	151 (35.1)	0.008
Hypertension, *n* (%)	44 (48.9)	235 (54.7)	0.319
Diabetes, *n* (%)	31 (34.4)	116 (27.0)	0.153
History of heart disease (chronic heart failure excluded), *n* (%)	38 (42.2)	116 (27.0)	0.004
Cerebrovascular disease, *n* (%)	44 (48.9)	196 (45.6)	0.567
Respiratory disease, *n* (%)	40 (44.4)	50 (11.6)	0.001
Acute kidney injury, *n* (%)	5 (5.6)	5 (1.2)	0.019
Valvular heart disease, *n* (%)	2 (2.2)	26 (6.0)	0.140
Pericardial effusion, *n* (%)	4 (4.4)	19 (4.4)	0.991
Pulmonary hypertension, *n* (%)	8 (8.9)	35 (8.1)	0.814
Cognitive impairment, *n* (%)	4 (4.4)	6 (1.4)	0.055

*BMI* body mass index, *ACCI* age-adjusted Charlson comorbidity index, *HB* hemoglobin

Values are mean ± standard deviation or number (percentage) as appropriate. *P* < 0.05, statistical significance. In this study, anemia was defined as hemoglobin level < 120 g/L for males and < 110 g/L for females; Hypoproteinemia refers to serum albumin less than 35 g/L

**Table 2:** Perioperative parameters of patients

Variables	Heart failure group	Non-heart failure group	*P*
*Preoperative waiting time, n (%)*			0.028
≤ 2 day	14 (15.6)	30 (7.0)	
> 2 day	74 (84.4)	400 (93.0)	
ASA class ≥ 3, *n* (%)	66 (77.3)	67 (15.6)	0.001
*Anesthesia pattern, n (%)*			0.755
General anesthesia	53 (58.9)	267 (62.1)	
Local anesthesia	27 (30.0)	125 (29.1)	
Compound anesthesia	10 (11.1)	38 (8.8)	
Operation time ≥ 2 h, *n* (%)	21 (23.3)	119 (27.7)	0.398
HB at admission	110.1 ± 15.9	114.4 ± 16.6	0.023
Albumin at admission	35.8 ± 3.7	35.51 ± 5.0	0.219
CK at admission	163.2 ± 250.7	139.2 ± 152.7	0.209
BNP at admission	83.1 ± 60.9	72.3 ± 79.0	0.001
cTn I at admission	0.05 ± 0.01	0.05 ± 0.03	0.105
Serum potassium at admission	3.9 ± 0.5	3.9 ± 1.7	0.035
Serum sodium at admission	137.7 ± 4.3	137.9 ± 3.2	0.619
Creatinine value at admission	69.6 ± 22.1	72.05 ± 41.1	0.446
Left ventricular ejection fraction	62.1 ± 5.9	63.39 ± 5.1	0.031
Blood transfusion before operation, *n* (%)	36 (40.0)	115 (26.7)	0.012
Intraoperative blood transfusion, *n* (%)	19 (21.1)	70 (16.3)	0.268

*ASA* American Society of Anesthesiologists classification, *CK* creatine kinase, *BNP* B-type natriuretic peptide, *cTn I* Cardiac troponin I

Values are mean ± standard deviation or number (percentage) as appropriate. *P* < 0.05, statistical significance

**Table 3:** Independent predictors of perioperative acute heart failure in the multivariate analysis

Variables	Regression coefficient	Standard error	Wald *χ*^2^ value	OR (95%CI)	*P*
Respiratory diseases	2.039	0.356	32.788	7.68 (3.82, 15.43)	0.001
History of heart disease (chronic heart failure excluded)	0.792	0.318	6.191	2.21 (1.18, 4.12)	0.010
ASA class ≥ 3	2.671	0.316	71.337	14.46 (7.78, 26.87)	0.001
Preoperative waiting time ≤ 2 day	1.200	0.467	6.599	3.32 (1.33, 8.30)	0.010

*95% CI* 95% confidence interval, *OR* odds ratio, *ASA* American Society of Anesthesiologists classification

**Table 4:** ROC curve analysis results of statistically significant continuous variables

Variables	AUC (95% CI)	Cut­off value	Youden index	Standard error	Sensitivity	Specificity	*P*
Age (years)	0.595 (0.534, 0.656)	76.0	0.170	0.031	76.70	40.30	0.005
HB at admission	0.420 (0.357, 0.483)	75.25 g/L	0.012	0.032	98.90	2.30	0.017
BNP at admission	0.621 (0.564, 0.677)	32.5 pg/nl	0.256	0.029	87.80	37.80	0.001
LVEF	0.427 (0.360, 0.494)	73.5%	0.028	0.034	4.4	98.4	0.029
ACCI	0.619 (0.558, 0.680)	4.500	0.188	0.031	67.80	51.00	0.001

*HB* hemoglobin, *BNP* B-type natriuretic peptide, *LVEF* left ventricular ejection fraction, *ACCI* age-adjusted Charlson comorbidity index, *AUC* the area under the curve, *95% CI* 95% confidence interval

### Prediction model construction

Based on the above results of multivariate logistic analysis, we built a prediction model, *Z* =  − 5.964 + 2.039 × (Respiratory disease) + 0.792 × (history of heart disease (chronic heart failure excluded)) + 2.671 × (ASA class ≥ 3) + 1.200 × (Preoperative waiting time ≤ 2 days).

**Fig. 2:**
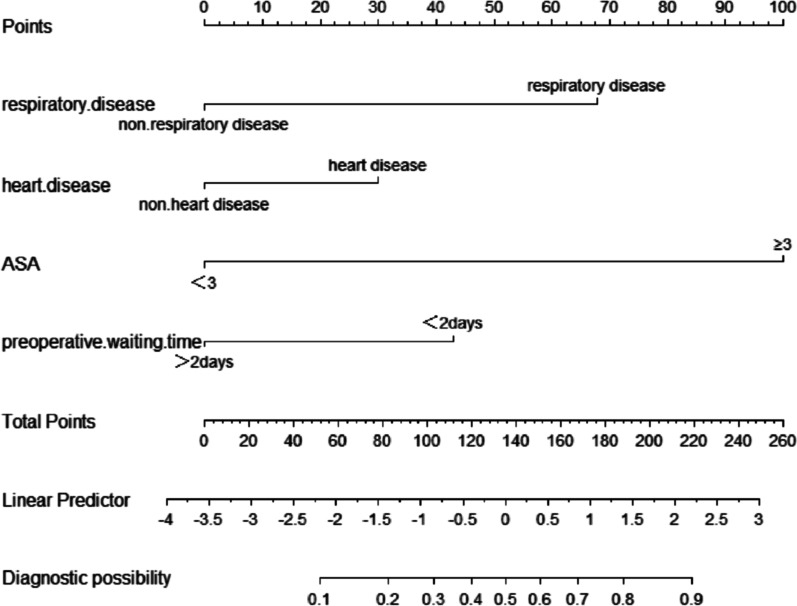
The nomogram of the risk of perioperative acute heart failure in older hip fracture patients. In this study, heart disease refer to history of heart disease (chronic heart failure excluded)

**Fig. 3:**
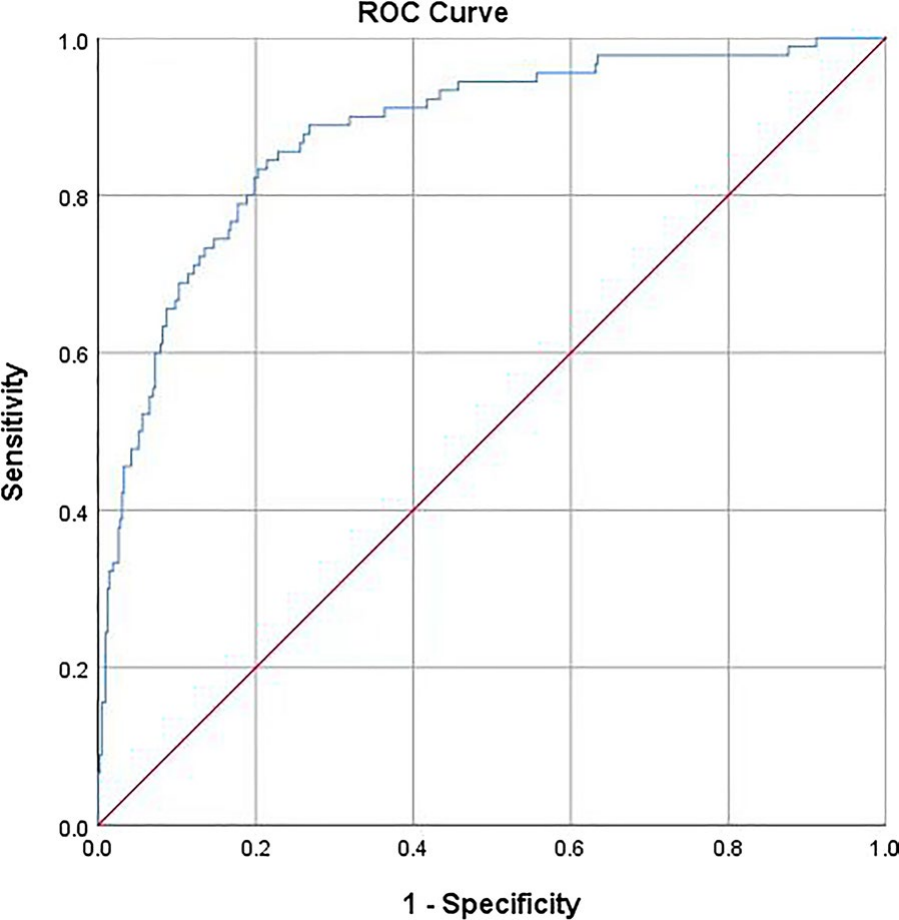
Receiver operating characteristic curve of the modeling group

**Fig. 4:**
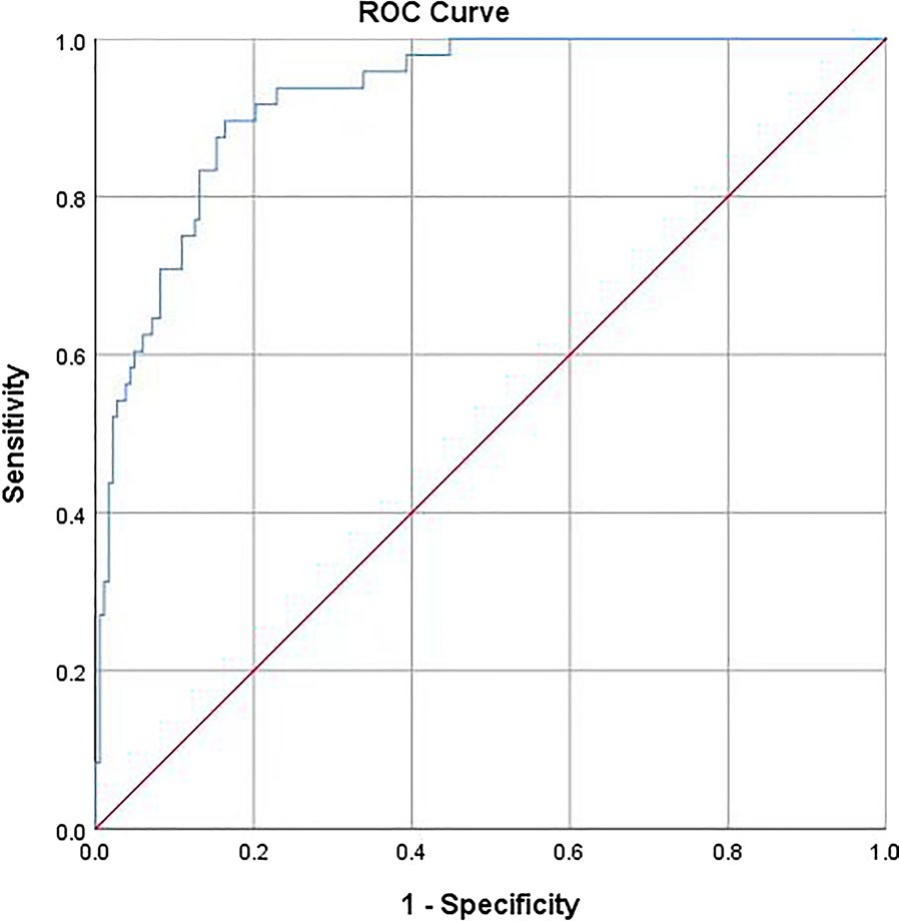
Receiver operating characteristic curve of the validation group

Figure 2 shows a nomogram of the risk of perioperative AHF in older hip fracture patients. According to the classification of variables in the nomogram, the scores corresponding to each index can be obtained and the total score can be calculated by adding the scores. The prediction probability corresponding to the total score is the probability of perioperative AHF. Figures 3 and 4 represent the receiver operating characteristic (ROC) curve of the prediction model in the modeling group and validation group, respectively.

### Validation of the prediction model

A total of 231 older hip fracture patients who met the inclusion and exclusion criteria were used as the external validation group. The efficacy of model validation was verified through the external data set. As shown in Table 5, there was no significant difference between the modeling group and the validation group in the comparison of the baseline data (*P* > 0.05). To some certain extent, it ensures the homogeneity of the two groups in the selection of research objects and the reliability of the study.

**Table 5:** Comparison of baseline data between modeling group and validation group

Variables	Modeling group (*n* = 520)	Validation group (*n* = 231)	*P*
Age (years)	78.9 ± 7.6	79.3 ± 7.7	0.559
*Gender, n (%)*			0.844
Male	136 (26.2)	62 (26.8)	
Female	384 (73.8)	191 (73.2)	
*Fracture site, n (%)*			0.240
Intertrochanteric fracture	264 (50.8)	128 (55.4)	
Femoral neck fracture	256 (49.2)	103 (44.6)	
*Number of complications* ≥ *3, n (%)*			0.218
Yes	218 (41.9)	108 (46.8)	
No	302 (58.1)	123 (53.2)	

Values are mean ± standard deviation or number (percentage) as appropriate. *P* < 0.05, statistical significance

## Discussion

AHF is a common and serious complication in older patients with hip fractures. In this study, we found that the prevalence of AHF was 18.37%. The results demonstrate that respiratory disease, history of heart disease (chronic heart failure excluded), ASA ≥ 3, and preoperative waiting time ≤ 2 days were independent risk factors for perioperative AHF in older hip fracture patients.

The AUC of the risk prediction model constructed was 0.877 in the modeling group and 0.910 in the validation group. It can be seen that the prediction model is highly accurate in identifying the occurrence of perioperative AHF. Regarding the Hosmer–Lemeshow test, the *P* values were all greater than 0.05, indicating that the degree of calibration of the prediction model is good. The nomograph drawn based on the model visualizes the risk and makes the model more scientific and practical.

The worse the patient's health condition before injury, the more likely they will have perioperative AHF, especially the patients with respiratory diseases and cardiac history. Paul found that the chance of heart failure in patients with chronic obstructive pulmonary disease (COPD) is 2.17 times high than in those without COPD, which is close to 1.9 times that of Truls et al. [21, 22]. Patients with COPD often have decreased lung function and increased lung volume, which may lead to myocardial injury and left ventricular diastolic dysfunction, inducing the occurrence of AHF [23, 24]. In this study, patients with a history of heart disease (chronic heart failure excluded) have a 2.21-fold increased risk of AHF, compared to patients without heart disease before injury. Currently, the contribution of heart disease to the AHF is explained by volume overload. Cardiovascular disease can lead to the weakening of cardiac pumping functions and fibrosis of the myocardial structure, increasing the risk of AHF [25]. For patients with cardiopulmonary insufficiency, airway management, and volume management should be strengthened to stabilize the internal environment. In our institution, we encourage patients to maintain the patency of the airway through effective coughing and deep breathing, and on the other hand, clinicians should adjust the rehydration plan timely to keep the circulation volume consistent with cardiovascular function.

In this study, we found that ASA ≥ 3 was the strongest predictor of perioperative AHF in older hip fracture patients, with an OR of 14.46 as compared with those with lower ASA class. ASA grading standard is a scoring system that can be used to assess patients’ operative risk of patients and guide resource allocations [26, 27]. In addition, a higher ASA class was demonstrated to be associated with a higher probability of pulmonary embolism, myocardial infarction, and heart failure, and a poorer health condition and operative tolerance [13, 28]. Given that, orthopedic surgeons and anesthesiologists should jointly conduct preoperative visits and anesthesia risk assessments for patients with high ASA scores.

The optimal timing of surgery for older patients with hip fracture has still been controversial. Relevant guidelines from abroad recommend that patients with hip fractures should receive early aggressive surgical treatment within 48 or even 24 h after injury [29, 30]. However, some researchers found that early surgical intervention in medically unstable patients can increase the risk of mortality [31], which could partially explain the finding of this study that patients with early surgical treatment (< 48 h) have a relative risk of AHF of 3.32, compared with those with ≥ 48 h of preoperative wait. Additionally, the quality of preoperative preparation may affect the prognosis of the patients. The older adults usually have multiple comorbidities, thus requiring more time to optimize clinical conditions to better tolerate the upcoming operation. However, scholars have inconsistent views on whether the delay of surgery will increase the risk of complications [32–34], with different or specific medical environments that could result in variable therapeutic effects of early surgery. On the other hand, affected by the allocation of medical resources, it is difficult for most hospitals to perform surgery within 48 h after injury in China. Therefore, how to choose the operation time, especially optimal timing for patients with great clinical benefit, remains a concern. More attention should be paid to the preoperative optimization of medical conditions, not only limited by the specific time of early surgery. Especially, for hip fracture patients who have heart disease or respiratory disease and are in poor general condition, it is better to prepare the condition for surgery rather than perform surgery while the general condition is unstable.

Nomograms integrate multiple independent risk factors identified via the multivariate regression analysis, and assign a value according to the contribution of each risk factor to the outcome variable, proving an excellent results visualization tool. In this study, the prediction model includes four easily available risk factors. Clinicians only need to draw vertical lines according to a specific proportion to obtain the prediction probability of each variable and calculate the sum to obtain the final probability of risk. This model helps doctors monitor the risk of AHF regularly and guide medical personnel in correcting potential changeable risk factors, thereby facilitating the reduction of perioperative AHF.

The merits of this study were the establishment of a nomogram for visualized assessment of risk factors for perioperative AHF in older hip fracture patients. However, several limitations should be mentioned. First, there was a bias in the selection of subjects, because this study was a single-center retrospective study. Therefore, the results may have been affected by the inaccuracy of the collected data and the absence of external validation, requiring prospective multicenter studies to verify. Second, although a multivariate regression model was used to minimize confounders, there are still unknown or unmeasured confounders, such as the perioperative fluid balance condition, the perioperative defecation condition, etc. Third, the study sample was limited, thus having less power to detect the significance of some infrequent variables, such as renal failure, which was more likely associated with electrolyte disturbance and caused adverse cardiac events [35, 36]. Fourth, the relationship between the identified factors and the incidence of AHF was associative rather than causal, thus, it should be carefully interpreted. However, the causal relationship between preoperative preparation time and preoperative heart failure cannot be determined, which needs to be explored by further research.

## Conclusion

In summary, we observed that the overall incidence of perioperative AHF in older patients undergoing hip fracture surgery was 18.37%. Preoperative respiratory disease, history of heart disease (chronic heart failure excluded), preoperative preparation time ≤ 2 days, and ASA class ≥ 3 were the independent risk factors for perioperative AHF, and further forming the readable nomogram to facilitate its use in practice and, subsequently, the potential reduction of AHF. Future studies with prospective and multicenter designs are warranted to verify our findings.

## Data Availability

All the data used during the current study are available from the corresponding author on reasonable request.

## References

[1] Ushirozako H, Ohishi T, Fujita T, Suzuki D, Yamamoto K, Banno T (2017). Does N-terminal pro-brain type natriuretic peptide predict cardiac complications after hip fracture surgery?. Clin Orthop Relat Res.

[2] Cullen M, Gullerud R, Larson D, Melton L, Huddleston J (2011). Impact of heart failure on hip fracture outcomes: a population-based study. J Hosp Med.

[3] Patel A, Pavlou G, Mújica-Mota R, Toms A (2015). The epidemiology of revision total knee and hip arthroplasty in England and Wales: a comparative analysis with projections for the United States. A study using the National Joint Registry dataset. Bone Joint J..

[4] Maffulli N, Aicale R (2022). Proximal femoral fractures in the elderly: a few things to know, and some to forget. Medicina.

[5] Marsillo E, Pintore A, Asparago G, Oliva F, Maffulli N (2022). Cephalomedullary nailing for reverse oblique intertrochanteric fractures 31A3 (AO/OTA). Orthop Rev.

[6] Gargano G, Poeta N, Oliva F, Migliorini F, Maffulli N (2021). Zimmer Natural Nail and ELOS nails in pertrochanteric fractures. J Orthop Surg Res.

[7] Roche J, Wenn R, Sahota O, Moran C (2005). Effect of comorbidities and postoperative complications on mortality after hip fracture in elderly people: prospective observational cohort study. BMJ.

[8] Pareja Sierra T, Bartolomé Martín I, Rodríguez Solís J, Bárcena Goitiandia L, Torralba González de Suso M, Morales Sanz M (2017). Predictive factors of hospital stay, mortality and functional recovery after surgery for hip fracture in elderly patients. Rev Esp Cir Ortop Traumatol.

[9] Groff H, Kheir M, George J, Azboy I, Higuera C, Parvizi J (2020). Causes of in-hospital mortality after hip fractures in the elderly. Hip Int.

[10] Chatziravdeli V, Vasiliadis A, Vazakidis P, Tsatlidou M, Katsaras G, Beletsiotis A (2021). The financial burden of delayed hip fracture surgery: a single-center experience. Cureus.

[11] Açan A, Gültaç E, Kılınç C, Özlek B, Gürsan O, Biteker M (2020). Preoperative mild pericardial effusion is associated with perioperative complications in elderly patients following hip fracture surgery. J Invest Surg.

[12] You F, Ma C, Sun F, Liu L, Zhong X (2021). The risk factors of heart failure in elderly patients with hip fracture: what should we care. BMC Musculoskelet Disord.

[13] Meyer A, Eklund H, Hedström M, Modig K (2021). The ASA score predicts infections, cardiovascular complications, and hospital readmissions after hip fracture—a nationwide cohort study. Osteoporos Int.

[14] Chen YP, Kuo YJ, Hung SW, Wen TW, Chien PC, Chiang MH (2021). Loss of skeletal muscle mass can be predicted by sarcopenia and reflects poor functional recovery at one year after surgery for geriatric hip fractures. Injury.

[15] Quaranta M, Miranda L, Oliva F, Migliorini F, Pezzuti G, Maffulli N (2021). Haemoglobin and transfusions in elderly patients with hip fractures: the effect of a dedicated orthogeriatrician. J Orthop Surg Res.

[16] Williams B, Doddamani S, Troup M, Mowery A, Kline C, Gerringer J (2017). Agreement between heart failure patients and providers in assessing New York Heart Association functional class. Heart Lung.

[17] Blacher M, Zimerman A, Engster P, Grespan E, Polanczyk C, Rover M (2021). Revisiting heart failure assessment based on objective measures in NYHA functional classes I and II. Heart.

[18] Dupuis J, Nathan H, Wynands J (1991). Clinical application of cardiac risk indices: how to avoid misleading numbers. Can J Anaesth.

[19] Hernandez A, Newby L, O'Connor C (2004). Preoperative evaluation for major noncardiac surgery: focusing on heart failure. Arch Intern Med.

[20] McDonagh T, Metra M, Adamo M, Gardner R, Baumbach A, Böhm M (2021). 2021 ESC Guidelines for the diagnosis and treatment of acute and chronic heart failure. Eur Heart J.

[21] Carter P, Lagan J, Fortune C, Bhatt D, Vestbo J, Niven R (2019). Association of cardiovascular disease with respiratory disease. J Am Coll Cardiol.

[22] Ingebrigtsen T, Marott J, Vestbo J, Nordestgaard B, Lange P (2020). Coronary heart disease and heart failure in asthma, COPD and asthma-COPD overlap. BMJ Open Respir Res.

[23] Aisanov Z, Khaltaev N (2020). Management of cardiovascular comorbidities in chronic obstructive pulmonary disease patients. J Thorac Dis.

[24] Cherneva Z, Valev D, Youroukova V, Cherneva R (2021). Left ventricular diastolic dysfunction in non-severe chronic obstructive pulmonary disease—a step forward in cardiovascular comorbidome. PLoS ONE.

[25] Norring-Agerskov D, Madsen C, Bathum L, Pedersen O, Lauritzen J, Jørgensen N (2019). History of cardiovascular disease and cardiovascular biomarkers are associated with 30-day mortality in patients with hip fracture. Osteoporos Int.

[26] Irlbeck T, Zwißler B, Bauer A (2017). ASA classification: transition in the course of time and depiction in the literature. Anaesthesist.

[27] Kastanis G, Topalidou A, Alpantaki K, Rosiadis M, Balalis K (2016). Is the ASA score in geriatric hip fractures a predictive factor for complications and readmission?. Scientifica.

[28] Chen L, Liang J, Chen M, Wu C, Cheng H, Wang H (2017). The relationship between preoperative American Society of Anesthesiologists Physical Status Classification scores and functional recovery following hip-fracture surgery. BMC Musculoskelet Disord.

[29] Brox W, Roberts K, Taksali S, Wright D, Wixted J, Tubb C (2015). The American Academy of Orthopaedic Surgeons evidence-based guideline on management of hip fractures in the elderly. J Bone Joint Surg Am.

[30] Watanabe Y, Matsushita T (2005). Management of hip fracture in older people: a clinical guideline in Japan. Nihon Rinsho.

[31] Yonezawa T, Yamazaki K, Atsumi T, Obara S (2009). Influence of the timing of surgery on mortality and activity of hip fracture in elderly patients. J Orthop Sci.

[32] Mariconda M, Costa G, Cerbasi S, Recano P, Aitanti E, Gambacorta M (2015). The determinants of mortality and morbidity during the year following fracture of the hip: a prospective study. Bone Joint J.

[33] Simunovic N, Devereaux P, Sprague S, Guyatt G, Schemitsch E, Debeer J (2010). Effect of early surgery after hip fracture on mortality and complications: systematic review and meta-analysis. CMAJ.

[34] Saul D, Riekenberg J, Ammon J, Hoffmann D, Sehmisch S (2019). Hip fractures: therapy, timing, and complication spectrum. Orthop Surg.

[35] Schefold J, Filippatos G, Hasenfuss G, Anker S, von Haehling S (2016). Heart failure and kidney dysfunction: epidemiology, mechanisms and management. Nat Rev Nephrol.

[36] Zeisberg M, Koziolek M (2018). Cardio-renal axis: relationship of heart failure and renal insufficiency as comorbidities. Der Internist.

